# Profiling of host entry factors in adults and fetuses for severe acute respiratory syndrome coronavirus‐2 infection indicates its developmental regulation

**DOI:** 10.1002/ctm2.870

**Published:** 2022-07-08

**Authors:** Shizhe Yu, Haoren Wang, Lin Zhang, Qiang Cai, Duo Ma, Jian Zhao, Mengfei Ma, Zhiyong Yu, Zongping Xia

**Affiliations:** ^1^ Department of Clinical Systems Biology Laboratories The First Affiliated Hospital of Zhengzhou University Zhengzhou China; ^2^ Department of Hepatobiliary Surgery The Affiliated Hospital of Yunnan University Kunming China; ^3^ Department of Oncology The First Affiliated Hospital of Zhengzhou University Zhengzhou China; ^4^ Department of Cardiology The First Affiliated Hospital of Zhengzhou University Zhengzhou China; ^5^ Department of Neurology The First Affiliated Hospital of Zhengzhou University Zhengzhou China; ^6^ NHC Key Laboratory of Prevention and treatment of Cerebrovascular Diseases The First Affiliated Hospital of Zhengzhou University Zhengzhou China

Dear Editor,

Severe acute respiratory syndrome coronavirus 2 (SARS‐CoV‐2) is imposing the ongoing coronavirus disease 2019 (COVID‐19) pandemic. Despite numerous studies on the pathology of SARS‐CoV‐2 infection, we addressed the following three questions. (1) COVID‐19 manifests in respiratory, digestive, heart, cerebral, and urogenital tissues; however, there is heterogeneity regarding the virus loads and pathology among these organ systems within the same patient. (2) On one end of the COVID‐19 patient spectrum, fetuses would only display liver and renal injury due to possible maternal‐fetal vertical SARS‐CoV‐2 transmission in the third trimester of pregnancy. On the other end of the spectrum, adults are more susceptible to COVID‐19 and elderly patients are more prone to develop severe symptoms. Although multiple mechanisms might account for the observed discrepancies, differential expression of key entry‐related factors that mediate direct SARS‐CoV‐2 infection could be one underlying these two questions.^1^, ^2^ However, no study has systematically assessed the expression levels of virus entry‐related factors in fetuses and adults. (3) Angiotensin‐converting enzyme 2 (ACE2), the receptor mediating SARS‐CoV‐2 infection, and transmembrane serine protease 2 (TMPRSS2), the protease priming SARS‐CoV‐2 infection, is expressed in multiple cell types distributed across multiple tissues and organs; are their developmental relationships among the SARS‐CoV‐2 tropic cell types?

To answer the first two questions, we profiled and compared the expression patterns of ACE2, TMPRSS2 and neuropilin‐1 (NRP1) at both protein and messenger RNA (mRNA) levels across the whole human body tissues. NRP1 can mediate SARS‐CoV‐2 infection in a furin‐dependent manner when ACE2 expression is low or even in its absence if viral load is high, which might contribute to increased tropic cell types.^3^ For their protein expression patterns, we performed immunohistochemistry (IHC) and immunofluorescence colony (IFC) immunostaining against tissue microarray (TMA) specimens (Figure 1 and Figures S1 and S2). Our TMA specimens included 24 types of tissues from seven adult donors and 29 types of tissues from five fetuses (Table S2), which have the following uniqueness: each donor contributed multiple tissues, which enabled us to obtain reliable data about their expression levels for comparison. In addition, these donors were largely healthy and had not been exposed to SARS‐CoV‐2, therefore, the expression levels of the three factors were not affected by inflammatory and immune responses. For their mRNA, we analyzed a large cohort of publicly available single‐cell RNA sequencing (scRNA‐seq) datasets (Table S1). Upon IHC staining on the TMA specimens, their expression levels were evaluated based on histochemistry score (H‐score) quantification by HALO software (Figure 1A and Table S2). The H‐score bar plots revealed that: (1) ACE2 displayed weak expression in all the tissues examined from fetuses. (2) overall ACE2, TMPRSS2 and NRP1 displayed higher expression in adults than in fetuses, in which the difference of ACE2 was more notable between adult and fetal tissues, supporting adults were more susceptible to SARS‐CoV‐2 infection. (3) ACE2 displayed a wide range of expression levels among the different adult tissues examined, where the testis, intestine, and cerebrum were among the highest and the pancreas the lowest (Figure 1A,D,L,N). Specifically, for the respiratory tract, a relatively higher expression of ACE2 was observed in adults than in fetuses. However, its level in the lung was considerably low when compared to other adult tissues and was inconsistent with severe clinical manifestations in the lung. Notably, NRP1 expression in the lung was high and might play an important role in mediating direct virus infection (Figure 1A,H). ACE2 expression along the digestive tract was much higher in adults than in fetuses (Figures 1A–E, 3B and Figure S7A–C), whereas its expression in the liver and pancreas was similar between adults and fetuses (Figures 1A,F,G, 3B and Figure S7F). As to the heart, significant expression of ACE2, TMPRSS2 and NRP1 were observed in cardiac myocytes of adults, while no expression of ACE2 and NRP1 was detected in the fetal heart (Figure 1A,O). In addition, previous studies have reported that COVID‐19 patients aged over 60 were at higher risk of developing critical neurologic impairment.^4^ In the cerebrum, the expression of ACE2 and NRP1 was readily observed in endothelial and oligodendrocytes of adults but not in fetuses (Figure 1A,N and Figures S2I and S6).

**FIGURE 1:**
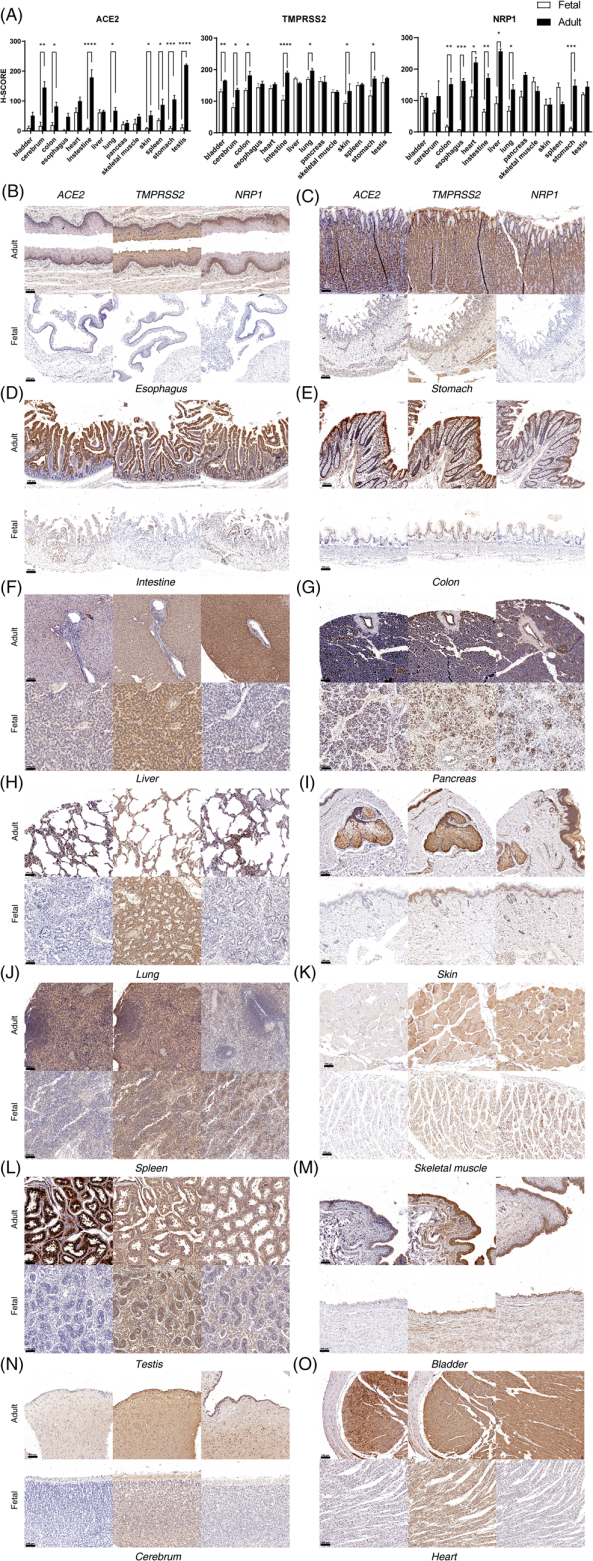
Tissue localization of angiotensin‐converting enzyme 2 (ACE2), transmembrane serine protease 2 (TMPRSS2), and neuropilin‐1 (NRP1) protein expression in paired healthy adult and fetal tissues. (A) Bar plots showing the H‐scores of ACE2/TMPRSS2/NRP1 protein expression in adult and fetal tissues. Error bars indicate scanning electron microscopy. Statistical significances were determined using the unpaired two‐tailed Student's *t*‐tests (*p < 0.05, **p < 0.005, ***p < 0.0005, ****p < 0.0001). (B–O) Representative immunohistochemistry staining of ACE2/TMPRSS2/NRP1 proteins in 14 adult tissues and 14 fetal tissues. For each tissue, three consecutive sections were stained using antibodies against ACE2, TMPRSS2, or NRP1 protein (brown), and counterstained with hematoxylin (blue), respectively

For the testis, our results found for the first time that there was no expression of ACE2 at the protein level in the fetal testis but a high level in adult testis (Figures 1L and 2D). Additionally, we analyzed and reconstructed the developmental trajectory of testes based on the integrated scRNA‐seq datasets representing neonates, children, youths, and adults (Figure S4A–E). ACE2 mRNA was mainly found in Sertoli cells, whereas TMPRSS2 was concentrated in cells undergoing the spermatogenesis process (Figure 2E). It is probably explicated the fact that no viral particles could be traced in the semen of infected patients (Figure 2D). Interestingly, we found that the ACE2 expression in Sertoli cells was gradually increased from the fetal stage throughout adulthood, which was positively correlated with the expression of the androgen receptor (AR) and anti‐müllerian hormone (AMH) (Figure 2A–C). Although more evidence is needed as to whether the virus can directly infect the testis, it is reasonable to suggest that SARS‐CoV2 could invade Sertoli cells, destroy the blood‐testis barrier, and cause testicular inflammation.^5^

**FIGURE 2:**
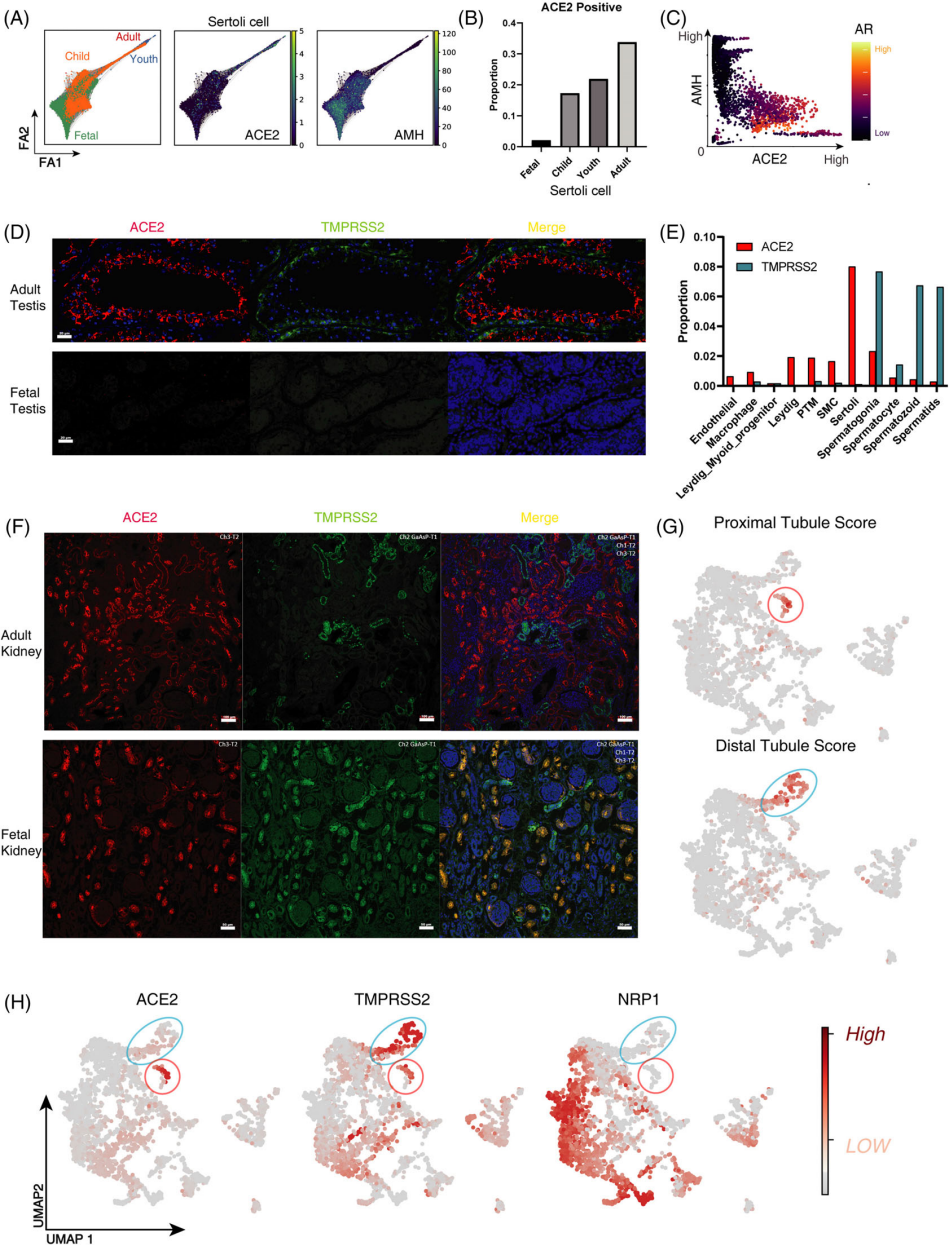
Characterization of the co‐expression patterns of ACE2/TMPRSS2/NRP1 in the male urogenital system. (A) Branching gene expression trajectory analysis showing the development trajectory of Sertoli lineage coloured by age stages and the expression of angiotensin‐converting enzyme 2 (ACE2) and AMH genes. (B) Bar plots of proportion of ACE2‐expressing Sertoli cells in the four age groups. (C) Scatter plot of AMH and ACE2 expression in Sertoli cells coloured by AR expression after MAGIC performed with the adaptive kernel. (D) IF analysis of ACE2 (the first column, red) and transmembrane serine protease 2 (TMPRSS2) (the second column, green) expression from adult and fetal testes. ACE2 is mainly detected in Sertoli cells while TMPRSS2 is mainly in spermatogonia cells. Superimposition revealed the absence of a co‐staining cell population. (E) Bar plots of proportion of ACE2‐ and TMPRSS2‐expressing cells in each cell type identified in Figure S4. (F) IF analysis of ACE2 (the first column, red), TMPRSS2 (the second column, green) from adult and fetal kidneys. ACE2 is mainly detected in proximal tubule cells while TMPRSS2 is mainly in distal tubule cells and collecting duct cells. Superimposition revealed the existence of a co‐staining cell population coloured yellow in the fetal kidney. (G–H) TSNE plots showing proximal tubule score, distal tubule score, and expression levels of ACE2/TMPRSS2/NRP1 in cells from fetal kidneys, which indicates that the ACE2/TMPRSS2 co‐expression cells are mainly enriched in fetal proximal tubule cells. The red circle represents the proximal tubule cells and the blue circle the distal tubule cells

Our IHC and IF results revealed high expression of ACE2 and TMPRSS2 in kidney specimens of adults and fetuses; however, cellular co‐localization of them could only be detected in fetal but not adult specimens (Figure S1G and Figure 2E).

Consistent with this, the scRNA‐seq analysis suggested that in fetal kidney both ACE2 and TMPRSS2 were expressed in proximal tubule cells, while TMPRSS2 was also expressed in distal tubules and collecting ducts (Figures 2F,G and 3B). In vitro differentiation and maturation model of kidney, organoids have also demonstrated the presence of a specific ACE2, TMPRSS2, and NRP1 triple positive stage in proximal tubule cells during the fetal kidney stage (Day 28) (Figure S5). The results suggest that the fetal kidney is highly susceptible to infection.

To answer the third question, we analyzed the Human Cell Landscape dataset, which included 702 968 cells from 118 samples covering 35 adult organs and 20 fetal organs (Figure S3).^6^ Cell type assignment revealed that ACE2 and TMPRSS2 were mainly expressed in epithelial cells, while NRP1 in stromal and immune cells (Figure 3A,B). Given most trophic cell types were those co‐expressing ACE2 and TMPRSS2, ACE2^+^TMPRSS2^+^ cells were extracted for developmental trajectory analysis (Figure 3C). The results demonstrated that the double‐positive cells were mainly differentiated from the same progenitor from endodermal cells. During early development, endodermal cells differentiate into the epithelia of the digestive tract and gland, respiratory tract, urinary system, and liver, which were highly consistent with susceptible tissues seen in clinical COVID‐19 patients.^7^

**FIGURE 3:**
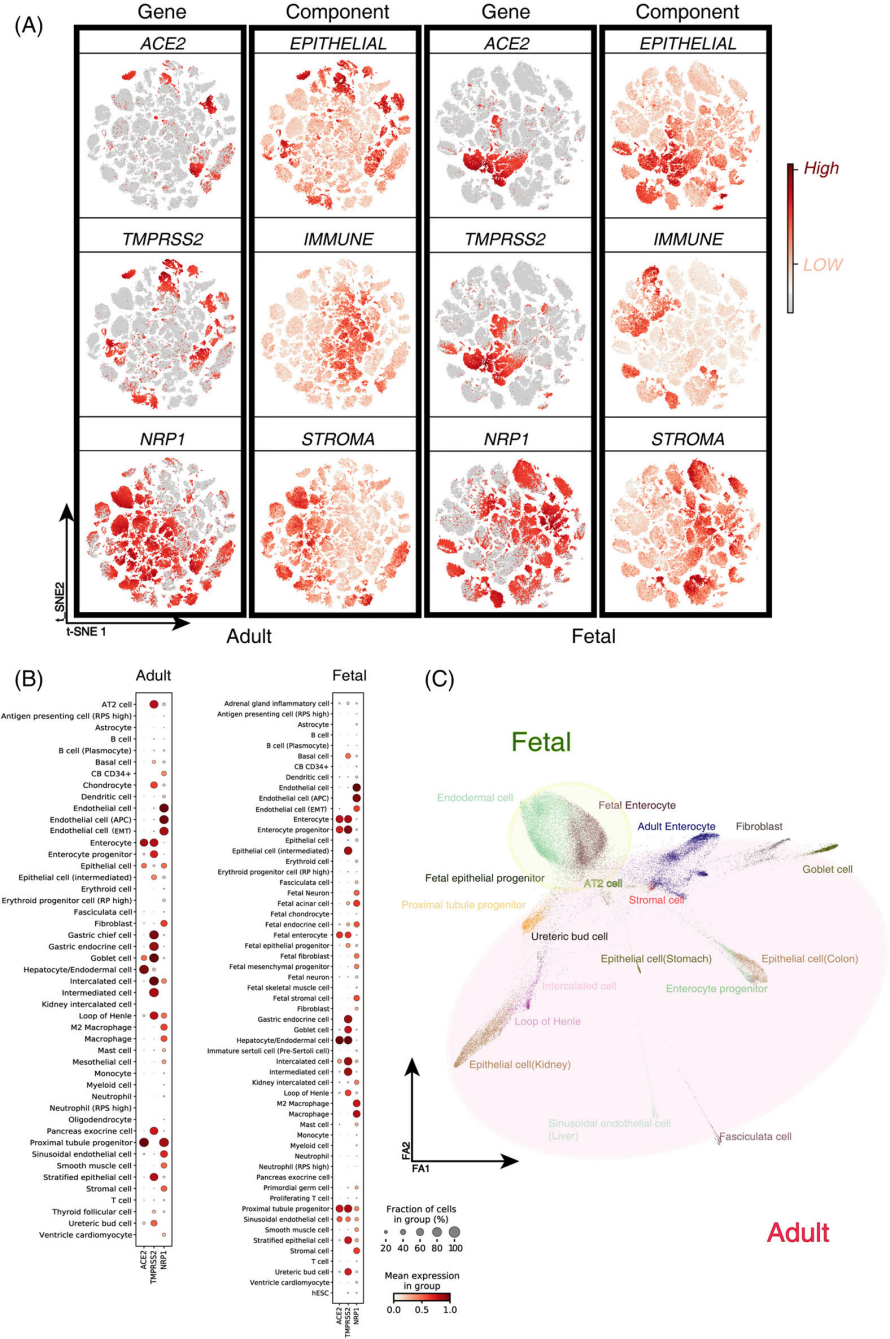
Single‐cell RNA sequencing (scRNA‐seq) based cell type characterization of angiotensin‐converting enzyme 2 (ACE2), transmembrane serine protease 2 (TMPRSS2), and neuropilin‐1 (NRP1) expression in human adult and fetal tissues TSNE plots showing the ACE2, TMPRSS2, and NRP1 expression levels of single cells. Epithelial score, Stroma score, and Immune score of all 702968 cells from adult and fetal tissues, which indicate the co‐expression cells, are mainly enriched in the epithelial compartment. RNA expression of ACE2/TMPRSS2/NRP1 from human cell landscape scRNA‐seq datasets across different adult and fetal cell types. For gene expression results in the dot plots, the dot size represents the proportion of cells within the respective cell type expressing the gene and the colour represents the average gene expression level within the particular cell type. Branching gene expression trajectory analysis of ACE2/TMPRSS2 double‐positive cells in fetal and adult human tissues using PAGA suggests the fetal to adult transition

In conclusion, our work demonstrated that the expression levels of SARS‐CoV‐2 entry‐related factors display substantial differences among different tissues and organs and between fetuses and adults, which may account for different SARS‐CoV‐2 susceptibility among different age groups and for different pathological manifestations among different tissues and organs. Our results also revealed that the major SARS‐CoV‐2 trophic cell types, although distributed across different tissues and organs, are probably all derived from endoderm, implying potential developmental regulation underlying the SARS‐CoV‐2 infection.

## Supporting information

FigureS1

FigureS2

FigureS3

FigureS4

FigureS5

FigureS6

FigureS7

Table S1 Information on the datasets included in this study.

Table S2 H‐scores of each protein in different tissues.

Table S3 Statistical comparisons between adults and fetuses.
