# Supplementary figures and images for: Profiling of host entry factors in adults and fetuses for severe acute respiratory syndrome coronavirus‐2 infection indicates its developmental regulation
Source: Clin Transl Med. 2022 Jul 8;12(7):e870. doi: 10.1002/ctm2.870 (PMC9270579; doi:10.1002/ctm2.870)

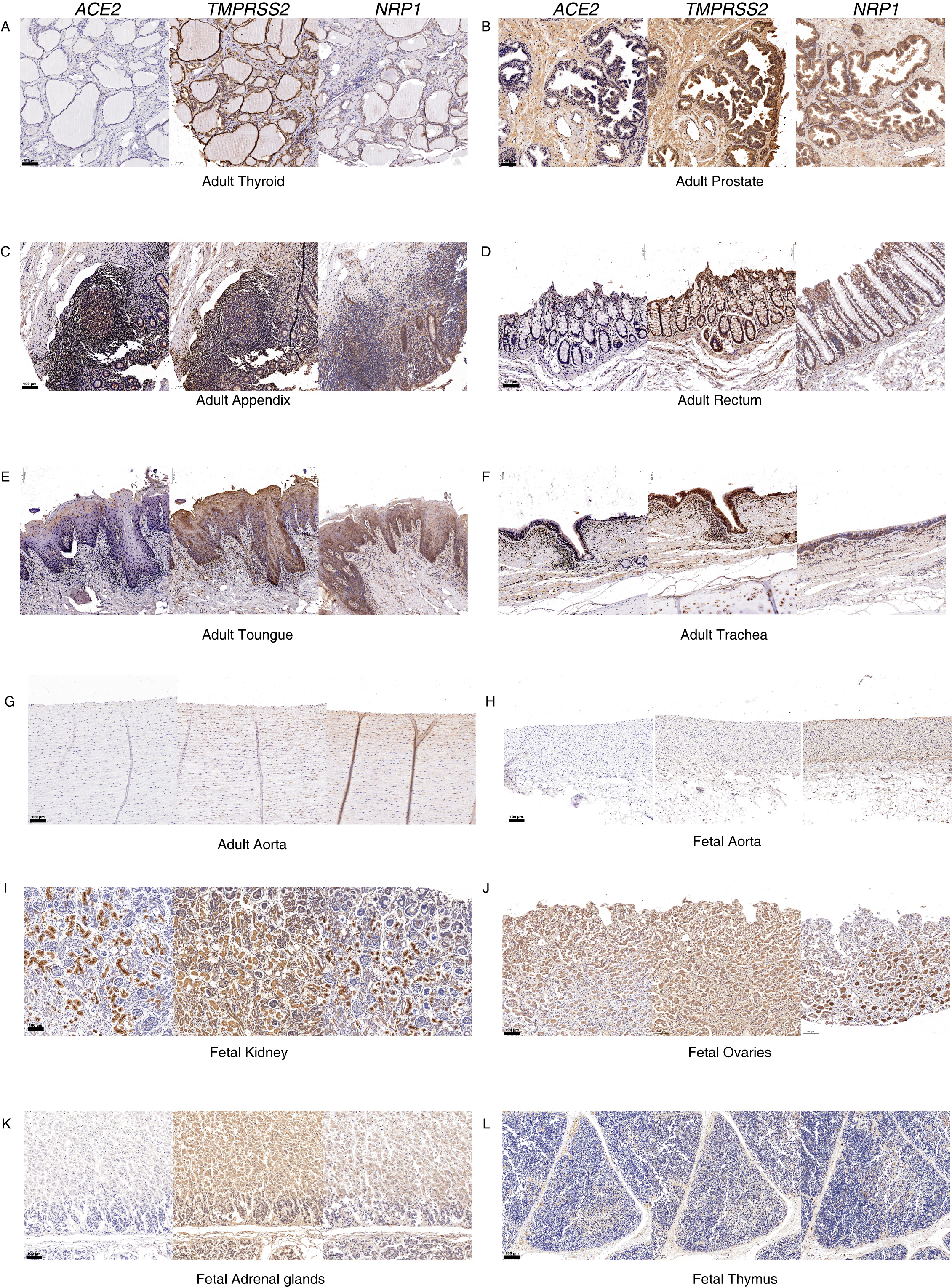

Supplement: Supplementary file 1 — FigureS1 [file CTM2-12-e870-s010.jpg]

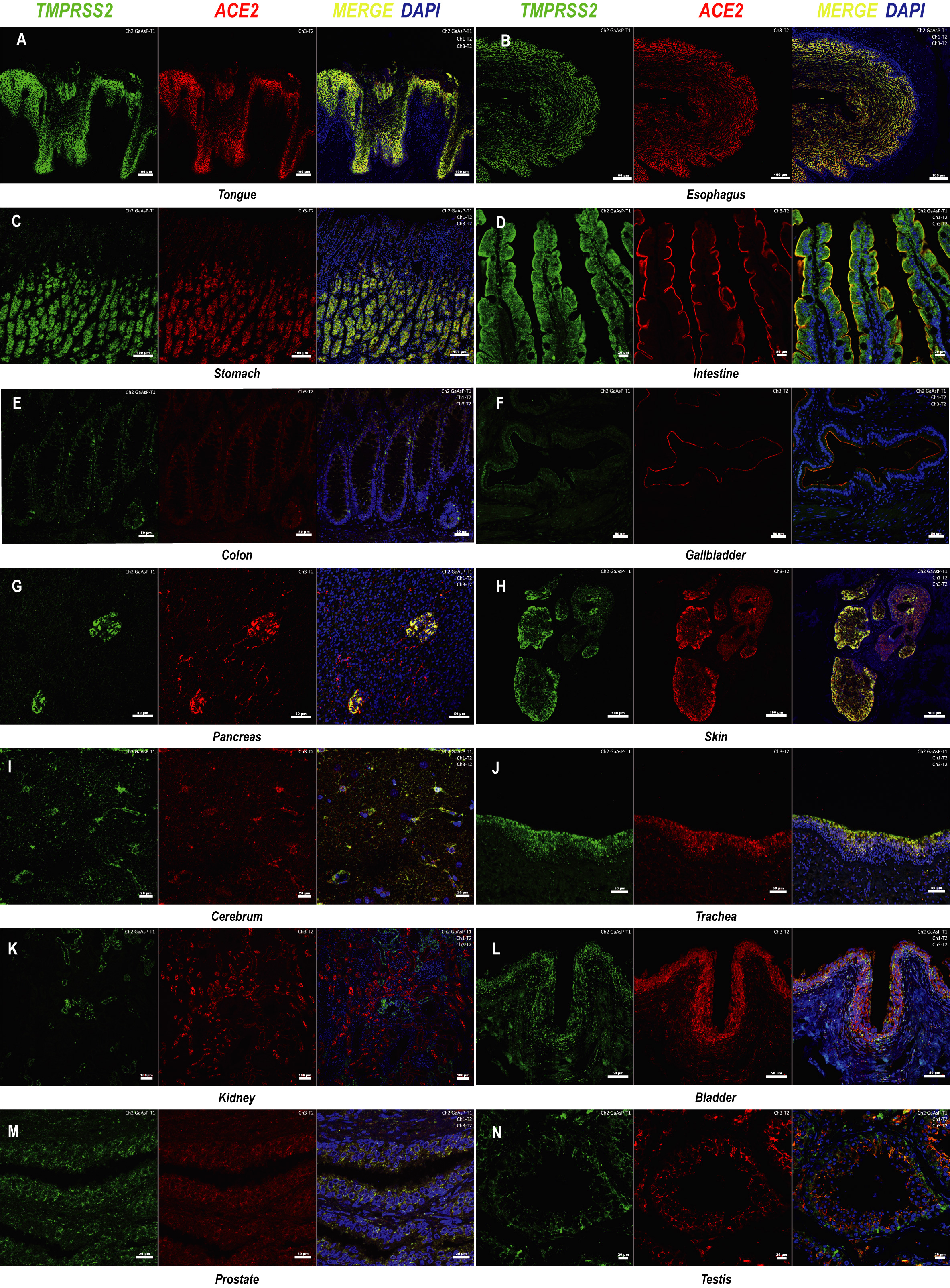

Supplement: Supplementary file 2 — FigureS2 [file CTM2-12-e870-s002.jpg]

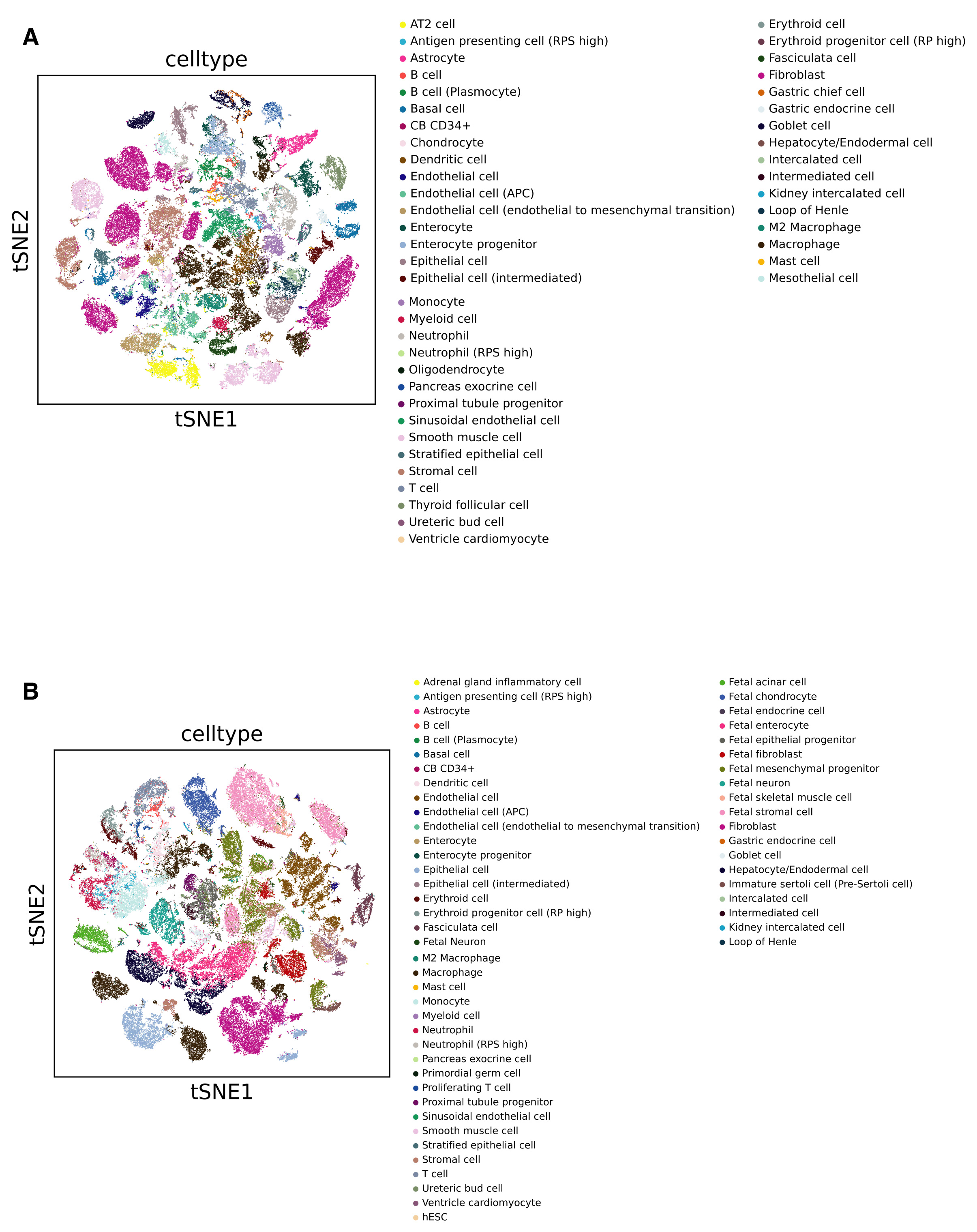

Supplement: Supplementary file 3 — FigureS3 [file CTM2-12-e870-s004.jpg]

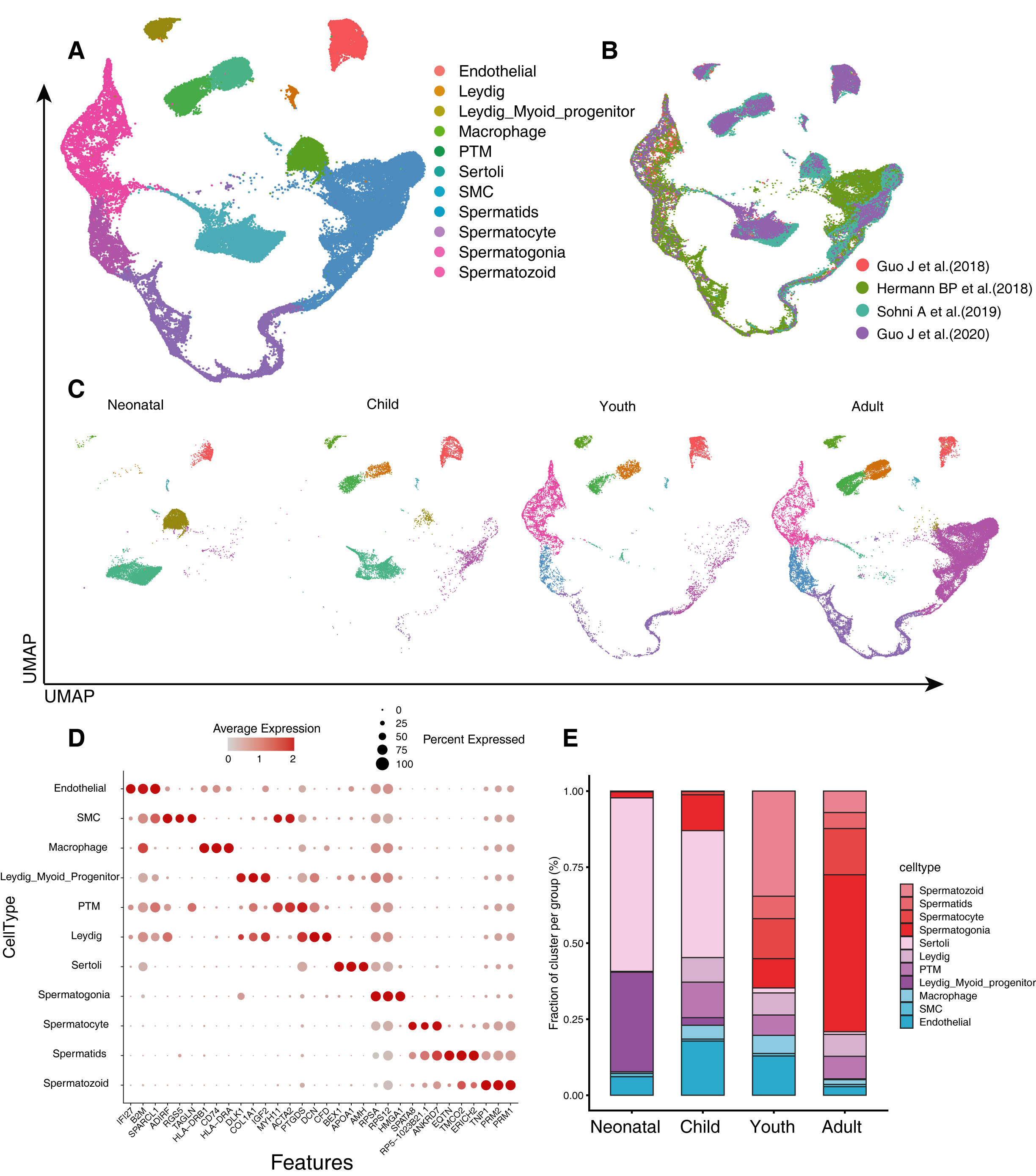

Supplement: Supplementary file 4 — FigureS4 [file CTM2-12-e870-s005.jpg]

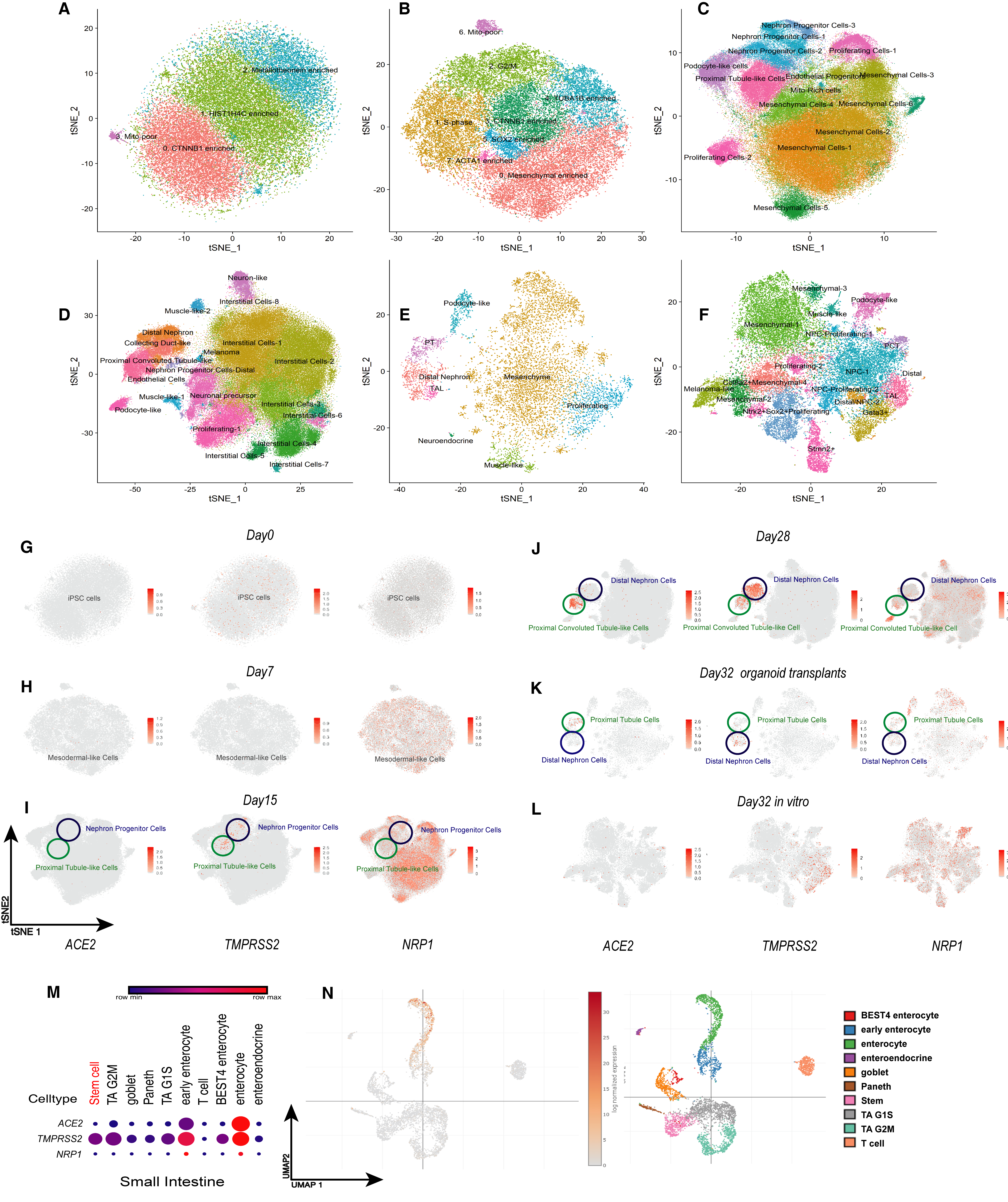

Supplement: Supplementary file 5 — FigureS5 [file CTM2-12-e870-s008.png]

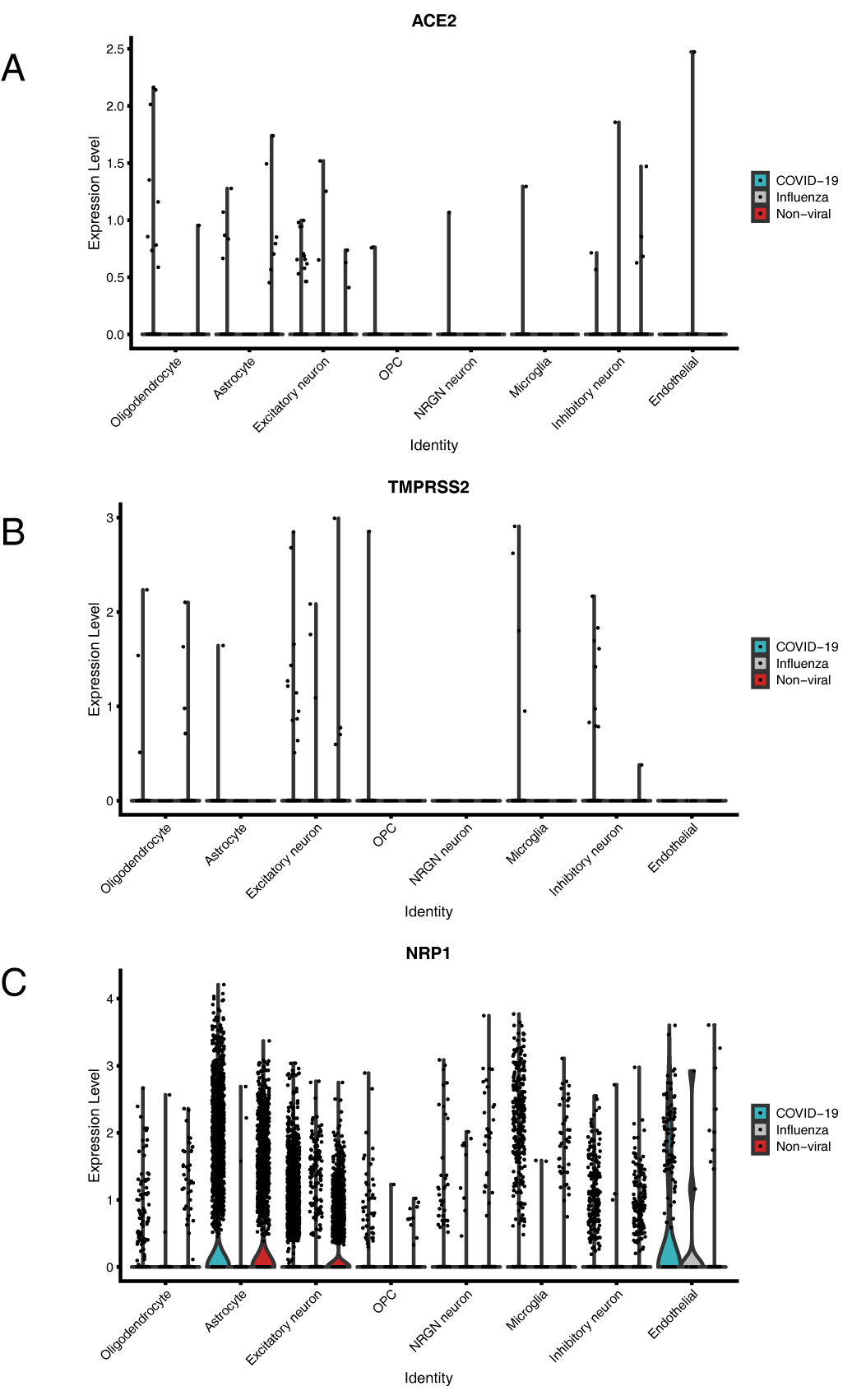

Supplement: Supplementary file 6 — FigureS6 [file CTM2-12-e870-s003.jpg]

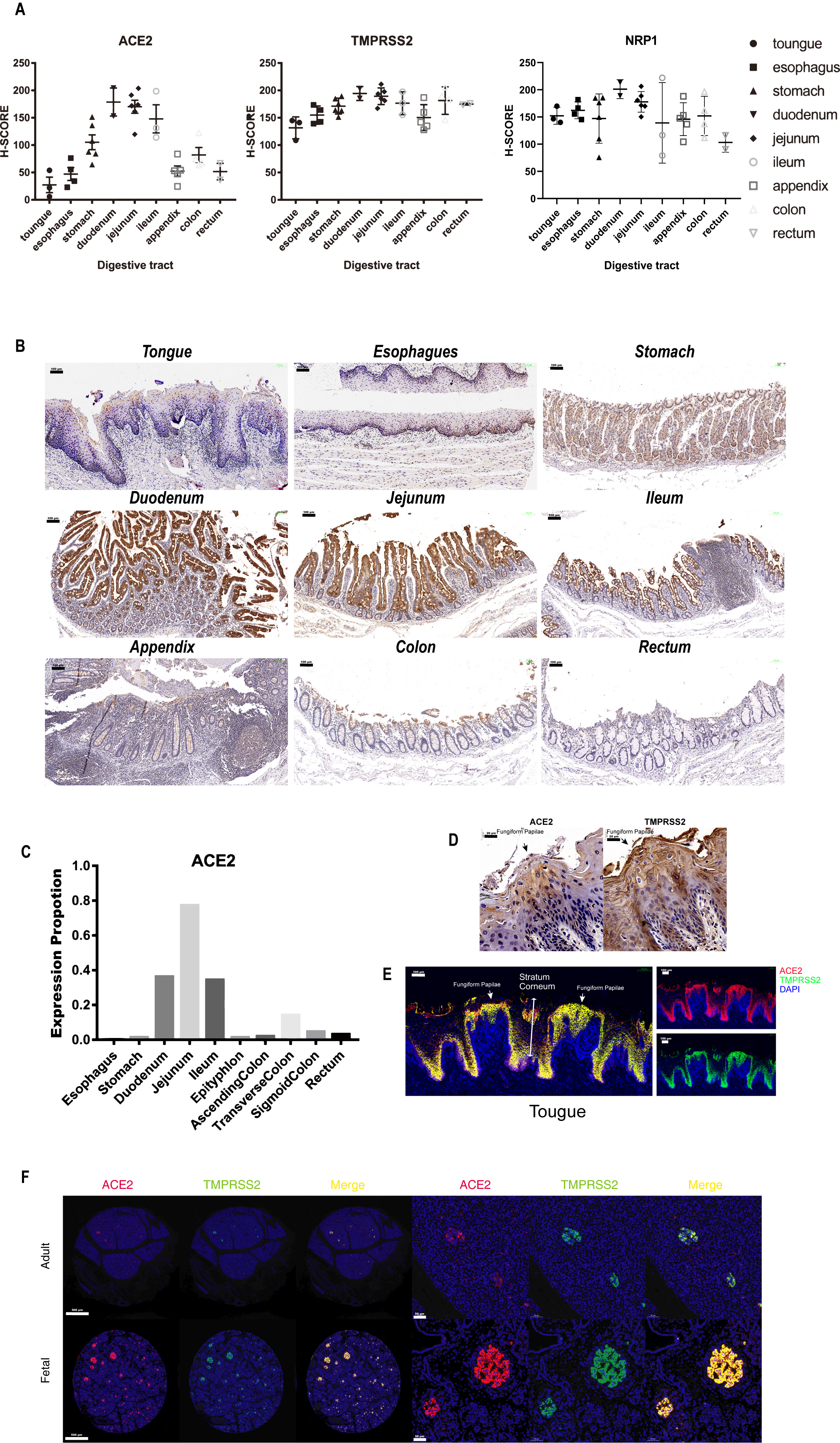

Supplement: Supplementary file 7 — FigureS7 [file CTM2-12-e870-s007.jpg]
